# Prediction of Lung Cancer Metastasis Using Machine Learning Models Based on Clinical Laboratory Data

**DOI:** 10.1002/cnr2.70350

**Published:** 2025-10-06

**Authors:** Chao Du, Qi Liu, Yuanyuan Guo, Jun Gong, Ling Yan, Zhijie Li, Changchun Niu

**Affiliations:** ^1^ Key Laboratory of Medical Laboratory Diagnostics of Education Ministry Chongqing Medical University Chongqing China; ^2^ Department of Laboratory Medicine Chongqing University Fuling Hospital Chongqing China; ^3^ Department of Laboratory Medicine Chongqing General Hospital, Chongqing University Chongqing China; ^4^ Information Technology Department People's Hospital of Chongqing Hechuan Chongqing China; ^5^ Chongqing Medical University Chongqing China

**Keywords:** laboratory data, lung cancer, machine learning, metastasis, regional lymph node involvement

## Abstract

**Background:**

Lymph node (N) or/and distant metastasis in lung cancer indicates poorer prognosis. While laboratory tests and computed tomography (CT) scans reflect tumor growth and metabolic activity, they usually require combination with other diagnostic methods to effectively assess metastasis, resulting in limited clinical use of these results.

**Aims:**

Develop machine learning models using diverse clinical laboratory data to predict lymph node invasion and skip N metastasis in lung cancer.

**Methods:**

This study performs regression analysis on lung cancer cases initially diagnosed by histopathology, categorized into N and M (skip N metastasis) groups by TNM stage. Laboratory and clinical test results were collected as characteristic parameters. Univariate analysis and lasso regression identified key predictors, and four machine learning algorithms developed the model.

**Results:**

Of the 1629 cases analyzed, 861 were assigned to the N group and 519 to the M group. Univariate analysis revealed significant differences in 40 parameters in Group N and 27 parameters in Group M (*p* < 0.05). LASSO regression identified 13 characteristic factors for the N group and 12 for the M group. In the N group, the factors included tumor size, prothrombin time (PT), mean platelet volume, fibrinogen, platelet count, procalcitonin, carbohydrate antigen 15–3 (CA 15–3), carcinoembryonic antigen (CEA), adenosine deaminase, red blood cell distribution width, thrombin time, smoking history, and alcohol consumption history. In the M group, the factors included cytokeratin 19 fragment, tumor size, CEA, CA 15–3, squamous cell carcinoma antigen (SCCA), alkaline phosphatase, fibrinogen, hemoglobin, calcium, albumin, PT, and absolute monocyte count. The test cohort results indicated that the logistic regression model was optimal for both groups, achieving AUC values of 0.888 and 0.875, respectively.

**Conclusion:**

The study demonstrated the potential of using ML algorithms, laboratory data, and clinical features to predict N involvement and skip N metastasis in lung cancer.

## 1 Introduction

Lung cancer is the leading cause of cancer‐related deaths globally [1, 2] and is also the most common malignant tumor in China [2]. Accurate staging is essential for improving survival rates; inaccurate staging, whether over‐ or under‐staging, can lead to ineffective treatments and potential harm to patients [3, 4]. Lymph node metastasis significantly impacts the prognosis of lung cancer [5]. When distant metastasis occurs, lung cancer is classified as stage IV, indicating a shift from potentially curable treatments to palliative care [3, 6]. Research indicates that the 5‐year survival rate for patients with early‐stage lung cancer (stage I) exceeds 68%, whereas for those with advanced stages (stages III‐IV), it drops below 36% [6]. Therefore, evaluating regional lymph node involvement and distant metastasis is crucial for devising the appropriate treatment plan and assessing prognosis.

Imaging examinations are commonly used as supportive tools for diagnosing and assessing lung cancer metastasis. Although various imaging techniques are available, differentiating between benign and malignant lesions remains a considerable challenge [7]. As lung cancer cells divide, the likelihood of their dissemination to lymph nodes increases [8, 9], potentially enabling cancer cells to enter the bloodstream via lymphatic vessel invasion and ultimately cause distant metastasis [10, 11]. In some cases, metastasis may bypass adjacent lymph nodes through alternative routes, leading to a special “skip metastasis” [12, 13], potentially advancing the cancer stage from I to IV. Therefore, evaluating local lymph node involvement is crucial for patient treatment decisions. Even when regional lymph nodes show no involvement, the potential for “skip metastasis” must be considered. This study aims to explore these two scenarios.

Machine learning (ML) algorithms are essential for diagnosing, treating, and predicting lung cancer outcomes using comprehensive and complex datasets [14]. Relevant studies in applied radiology and clinical laboratory data have been extensively reported. Inflammatory media [15, 16, 17], blood clotting disorders [18, 19], hematological parameters [15, 16, 20, 21], biochemical assays [22, 23], and other factors are associated with the development of lung cancer. Therefore, laboratory data can reflect the growth and metabolic activity of tumor cells.

Laboratory testing and low‐dose computed tomography (CT) imaging are typical protocols for patients being admitted for the first time. In the evaluation of lymph node involvement and distant metastasis in lung cancer, these tests frequently need to be combined with other diagnostic techniques for a comprehensive analysis. Assessing imaging parameters necessitates the extraction of various characteristic metrics and the engagement of qualified experts for interpretation [20, 24, 25, 26, 27, 28, 29, 30, 31, 32]. Consequently, this research aims to pinpoint an evaluation method that is both user‐friendly and precise, directly employing standard test outcomes. By merging ML algorithms with the findings from laboratory assessments and CT scans, this strategy establishes a dependable basis for clinical diagnosis and therapeutic approaches, allowing healthcare providers to create tailored treatment plans.

## 2 Materials and Methods

### 2.1 Inclusion and Exclusion Criteria for Cases

**FIGURE 1:**
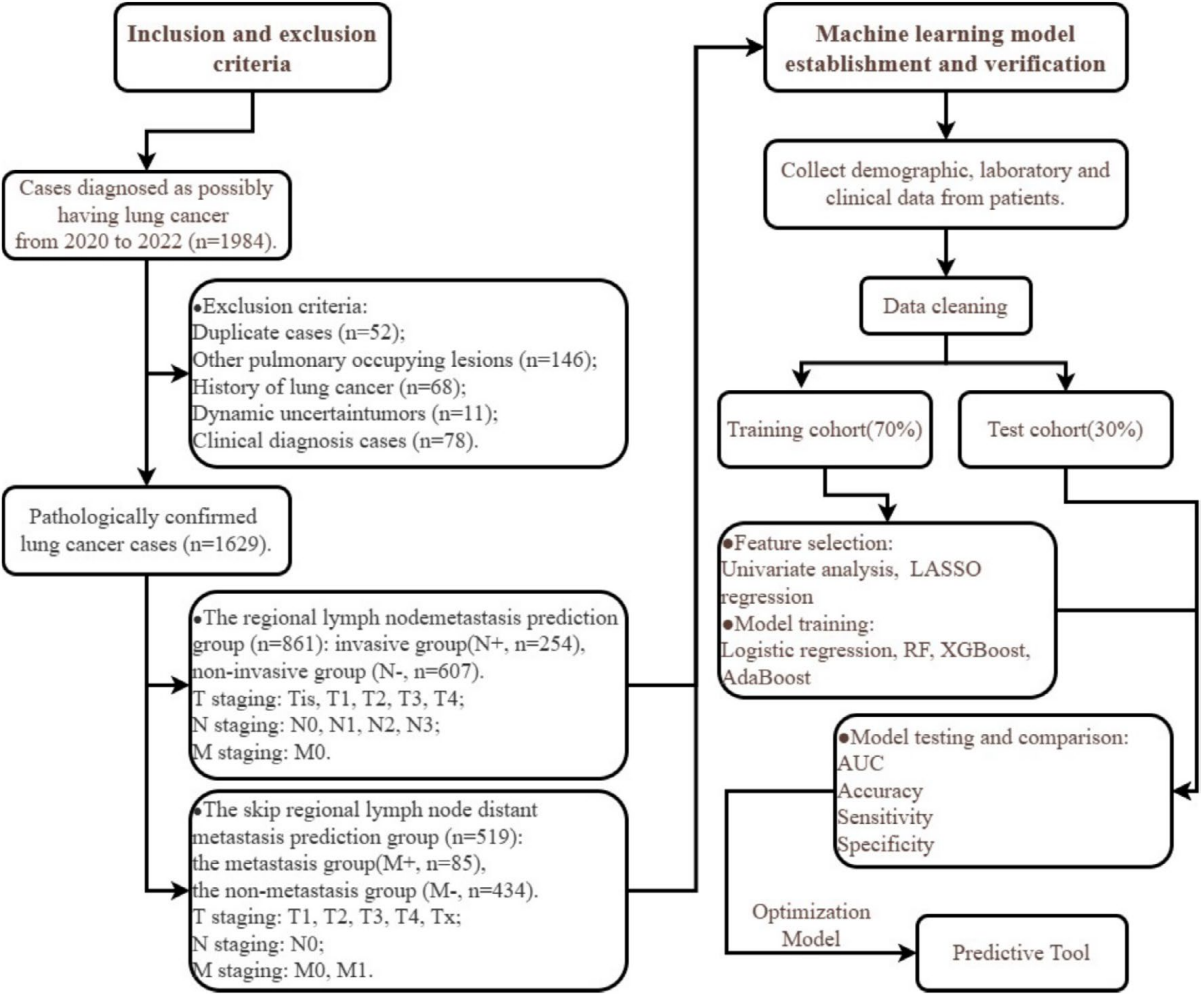
Case inclusion and exclusion criteria and data analysis flowchart. AdaBoost, adaptive boosting; RF, random forest; XGBoost, extreme gradient.

This retrospective study utilized cases from patients who were first diagnosed with lung cancer through histological examination (the pathological tissue comes from surgical resection or fine needle puncture) and had visited Chongqing University Fuling Hospital between January 2020 and December 2022. Cases were excluded if they had an unclear pathological diagnosis, a history of other malignancies, secondary lung malignancies, benign lung tumors, recurrent lung cancer post‐treatment, or had undergone lung cancer‐related treatment before diagnosis (Figure 1). The study was approved by the Ethics Committee of Chongqing University Fuling Hospital, with the approval number 2024CDFSFLYYEC‐053.

### 2.2 Collection of Patient Clinical Characteristics and Laboratory Data

The clinical characteristics included gender, age, height, weight, smoking and drinking history, tumor size, TNM stage, and pathological/histological results. For T staging in the TNM classification, the maximum diameter of solid nodules was measured by CT (unit: mm). For N staging, based on the results of postoperative or fine‐needle puncture lymphoid tissue, CT, positron‐emission tomography‐computed tomography (PET/CT), Magnetic Resonance Imaging (MRI), B‐ultrasound, and other imaging examinations, patients with suspected regional lymph node enlargement should receive 2–3 weeks of anti‐infective treatment to exclude possible infectious diseases, followed by re‐evaluation using imaging examinations. M staging was based on CT, PET/CT, MRI, B‐ultrasound, and other imaging findings.

The collected laboratory data include complete blood cell count, coagulation function tests, renal function tests, liver function tests, infection indices, tumor marker tests, blood calcium levels, serum glucose levels, and serum protein electrophoresis results. Whenever possible, all laboratory test parameters listed in the research report [20, 33, 34, 35, 36] should be collected.

### 2.3 Case Grouping

According to the included cases and the study purpose, patients were divided into two groups: the regional lymph node involvement prediction group (Group N) and the skip regional lymph node distant metastasis prediction group (Group M). Group N was further divided into non‐invasive group (N−) and invasive group (N+), with TNM stages of Tis‐4N0M0 and Tis‐4 N1‐3M0, respectively. Group M was divided into the non‐metastasis group (M−) and the metastasis group (M+), with TNM stages of T1‐4N0M0 and T1‐4N0M1, respectively. Finally, items with laboratory data missing by more than 30% in Groups N and M were excluded.

### 2.4 Statistical Analysis

Data preprocessing was executed using Excel 2013. Subsequent data analysis was performed using SPSS version 26.0 and R version 4.0.1. Categorical variables were reported as counts and percentages (*n* [%]), whereas continuous variables were depicted using medians and interquartile ranges (M [P25, P75]). For variables with missing data at a rate of less than 30%, imputation was conducted using the random forest algorithm. The Mann–Whitney *U* test was utilized for continuous variables that were not normally distributed, and the chi‐squared (*χ* ^2^) test was employed for categorical variable analysis. Feature selection was conducted with the least absolute shrinkage and selection operator (LASSO). ML models were developed using Logistic regression, random forest (RF), extreme gradient boosting (XGBoost), and adaptive boosting (AdaBoost), with the logistic model serving as a benchmark. The predictive performance of the models on the test cohort was assessed using the area under the curve (AUC) of the receiver operating characteristic (ROC) curve, accuracy (ACC), sensitivity (SEN), and specificity (SPE). The level of significance was set at *α* = 0.05.

## 3 Results

### 3.1 Case Enrollment

The research included 1629 patients diagnosed with lung cancer, with 861 individuals in Group N and 519 in Group M. Within Group N there were two subgroups: the lymph node involvement group (N+, *n* = 254) and the non‐lymph node involvement group (N−, *n* = 607). In Group N, there are two subgroups: the lymph node involvement group (N+, *n* = 254), with lymph node diameters ≥ 1 cm as determined by imaging examination; and the non‐lymph node involvement group (N−, *n* = 607), in which 575 cases were confirmed to be non‐invasive by postoperative lymph node pathology examination, while 33 cases were not found to be invasive by imaging examination after anti‐infection treatment; no distant metastasis was found in any of the 861 cases by imaging examination. In Group N, there were 667 cases of adenocarcinoma, 158 cases of squamous cell carcinoma, 27 cases of small cell lung cancer (SCLC), and 9 cases of other types.

Group M was divided into the metastasis group (M+, *n* = 85), in which distant metastasis was detected by imaging examination: 70 cases with one metastasis, 12 cases with two metastases, and 3 cases with more than three metastases. The metastatic sites included 44 cases of bone, 19 cases of pleura, 18 cases of brain, 10 cases of lung, 7 cases of kidney, 6 cases of liver, and 1 case of heart. In the non‐metastasis group (M−, *n* = 434), 402 cases were confirmed to have no regional lymph node invasion by postoperative lymph node pathology examination, 32 cases showed no metastasis by imaging examination after anti‐infection treatment, and no distant metastasis was found in any of the 434 cases by imaging evaluation. In group M, there were 443 cases of adenocarcinoma, 61 cases of squamous cell carcinoma, 7 cases of SCLC, and 8 cases of other types (Figure 1 and Table 1).

**TABLE 1:** Patient's TNM staging and pathological histological characteristics.

Clinical characteristics	Group N		Group M	
	N+	N−	M+	M−
	(*n* = 254)	(*n* = 607)	(*n* = 85)	(*n* = 434)
TNM classification:				
T classification				
Tis	0	173 (20.09%)	\	\
T1	32 (3.72%)	331 (38.44%)	20 (3.85%)	331 (63.78%)
T2	71 (8.25%)	71 (8.25%)	20 (3.85%)	71 (13.68%)
T3	67 (7.78%)	11 (1.28%)	11 (2.12%)	11 (2.12%)
T4	84 (9.76%)	21 (2.44%)	31 (5.97%)	21 (4.05%)
Tx	\	\	3 (0.58%)	0 (0%)
N classification				
N0	0	607 (70.5%)	85 (16.38%)	434 (83.62%)
N1	60 (6.97%)	0	\	\
N2	148 (17.19%)	0	\	\
N3	46 (5.34%)	0	\	\
M classification			\	\
M0	254 (29.5%)	607 (70.5%)	0 (0%)	434 (83.62%)
M1	\	\	85 (16.38%)	0 (0%)
Pathological histological type:				
Adenocarcinoma	116 (13.47%)	551 (64%)	65 (12.52%)	378 (72.83%)
Squamous cell carcinoma	109 (12.66%)	49 (5.69%)	12 (2.31%)	49 (9.44%)
SCLC	25 (2.9%)	2 (0.23%)	5 (0.96%)	2 (0.39%)
Others	4 (0.46%)	5 (0.58%)	3 (0.58%)	5 (0.96%)

Abbreviations: Group M, the skip regional lymph node distant metastasis prediction group; Group N, the regional lymph node involvement prediction group; M−, the non‐metastasis group; M+, the metastasis group; N−, non‐invasive group; N+, invasive group; SCLC, small cell lung cancer; TNM, tumor size, lymph node involvement, and distant metastasis.

### 3.2 Clinical Characteristics of Patients

Univariate analysis revealed that within the N group, age, gender, tumor size, BMI, smoking history, and drinking history exhibited statistically significant differences (*p* < 0.001). For the M group, age, tumor size, BMI, and smoking history also demonstrated significant differences (*p* < 0.05), whereas gender and drinking history did not (*p* > 0.05) (Table 2).

**TABLE 2:** Clinical characteristics of patients.

Clinical characteristics	Group N			Group M		
	N+	N−		M+	M−	
	(*n* = 254)	(*n* = 607)	*p*	(*n* = 85)	(*n* = 434)	*p*
Age (years)	67 (58, 73)	58 (52, 70)	< 0.001	69 (55, 76)	63 (54, 71.8)	0.001
Gender			< 0.001			0.073
Male	184 (21.37%)	258 (29.97%)		47 (9.06%)	194 (37.38%)	
Female	70 (8.13%)	349 (40.53%)		38 (7.32%)	240 (46.24%)	
Tumor size (mm)	43.6 (32,57)	14 (10,22)	< 0.001	38 (24.1,47)	17 (12,25)	< 0.001
BMI	22.39 (20.44,24.98)	23.6 (21.5,25.6)	< 0.001	22.7 (20.4,25.3)	23.5 (21.5,25.6)	0.018
Smoking history			< 0.001			0.002
Yes	158 (18.35%)	176 (20.44%)		42 (8.09%)	140 (26.97%)	
No	96 (11.15%)	431 (50.06%)		43 (8.29%)	294 (56.65%)	
Alcohol consumption history			< 0.001			0.07
Yes	93 (10.8%)	106 (12.31%)		25 (4.82%)	89 (17.15%)	
No	161 (18.7%)	501 (58.19%)		60 (11.56%)	345 (66.47%)	

Abbreviations: Group M, the skip regional lymph node distant metastasis prediction group; Group N, the regional lymph node involvement prediction group; M−, the non‐metastasis group.; M+, the metastasis group; N−, non‐invasive group; N+, invasive group.

### 3.3 Laboratory Data of Patients

Laboratory data has been collected in accordance with the above requirements. To ensure the model's performance and mitigate algorithmic deviation, the integrity rate of each data item after grouping must not be less than 30%. After excluding non‐conforming items, a total of 38 parameters were collected, with the missing rate of each parameter ranging from 0.12% to 19.86% in group N and from 0.19% to 21% in group M (Table 3). Missing data were imputed using the random forest algorithm.

**TABLE 3:** Collected laboratory parameters and missing rates.

Abbreviation	Parameter	Group N	Group M
ADA	Adenosine deaminase	1.86%	1.16%
ALB	Albumin	0.23%	0.19%
ALP	Alkaline phosphatase	0.35%	0.19%
ALPHA1	*α*1‐globulin ratio	3.48%	2.31%
ALPHA2	*α*2‐globulin ratio	3.48%	2.31%
APTT	Activated partial thromboplastin time	0.70%	0.58%
BASO#	Absolute basophil count	0.12%	0.19%
BETA1	*β*1‐globulin ratio	3.48%	2.31%
Ca	Calcium	0.58%	0.19%
CA153	Carbohydrate antigen 153	18.00%	20.62%
CEA	Carcinoembryonic antigen	12.54%	13.87%
CYFRA 211	Cytokeratin‐19 fragment	18.70%	20.62%
DBIL	Direct bilirubin	0.35%	0.19%
EO#	Absolute eosinophil count	0.12%	0.19%
Fib	Fibrinogen	0.58%	0.39%
GAMMA	*γ*‐globulin ratio	3.48%	2.31%
GLB	Globulin	0.46%	0.39%
GLU	Glucose	8.36%	9.25%
HCT	Hematocrit	0.12%	0.19%
HGB	Hemoglobin	0.12%	0.19%
LYMPH#	Absolute lymphocyte count	0.12%	0.19%
MCV	Mean corpuscular volume	0.12%	0.19%
MONO#	Absolute monocyte count	0.12%	0.19%
MPV	Mean platelet volume	1.16%	1.35%
NEUT#	Absolute neutrophil count	0.12%	0.19%
NSE	Neuron‐specific enolase	18.93%	20.81%
PCT^a^	Plateletcrit	1.16%	1.35%
PCT^b^	Procalcitonin	18.23%	20.23%
PLT	Platelet count	0.12%	0.19%
ProGRP	Pro‐gastrin‐releasing peptide	19.86%	21.00%
PT	Prothrombin time	0.70%	0.58%
RBC	Red blood cells	0.12%	0.19%
RDW	Red cell distribution width	0.12%	0.19%
SCCA	Squamous cell carcinoma‐related antigen	18.23%	20.42%
TBIL	Total bilirubin	0.35%	0.19%
TP	Total protein	0.35%	0.19%
TT	Thrombin time	0.70%	0.58%
WBC	White blood cell count	0.12%	0.19%

Continuous laboratory variables are displayed as median values in the quartile range (M [P25, P75]). Univariate analysis demonstrated that, within the N group, all laboratory parameters except GLU, BASO#, RBC, and ProGRP exhibited statistically significant intergroup differences (*p* < 0.05). In the M group, all laboratory variables except GLB, BETA1, GAMMA, ADA, DBIL, TBIL, GLU, BASO#, EO#, RBC, MCV, PLT, MPV, PCT^a^, and APTT displayed statistically significant intergroup disparities (*p* < 0.05) (Table 4).

**TABLE 4:** Laboratory data of patients.

Laboratory data	Group N			Group M		
	N+	N−	*p*	M+	M−	*p*
ADA (U/L)	9.7 (8.43, 11.92)	9.6 (7.8, 11.4)	0.019	9.8 (8, 12.4)	9.6 (7.9, 11.5)	0.384
ALB (g/L)	38.7 (35.1, 41.9)	42.5 (38.9, 44.8)	< 0.001	37 (33.3, 40.9)	42 (38.1, 44.3)	< 0.001
ALP (U/L)	81.5 (68.03, 95.95)	75.2 (61.6, 90.9)	< 0.001	86.1 (72108.2)	76.4 (61.9, 89.9)	< 0.001
ALPHA1 (%)	5 (3.8, 6.9)	3.8 (3.4, 5)	< 0.001	4.8 (4, 6.2)	3.9 (3.4, 5.3)	< 0.001
ALPHA2 (%)	9.85 (8.6, 11.49)	8.8 (7.9, 9.9)	< 0.001	9.9 (8.3, 11.3)	9 (8.1, 10.2)	0.001
APTT (s)	27.44 (25.6, 29.2)	26.9 (25.3, 28.6)	0.015	27.1 (24.9, 28.6)	26.8 (25.3, 28.5)	0.984
BASO# (10^9^/L)	0.03 (0.02, 0.04)	0	0.155	0.03 (0.02, 0.04)	0.03 (0.02, 0.04)	0.793
BETA1 (%)	6 (5.4, 6.5)	6.1 (5.7, 6.5)	0.004	6.1 (5.5, 6.4)	6.1 (5.7, 6.5)	0.137
Ca (mmol/L)	2.27 (2.18, 2.37)	2.3 (2.2, 2.4)	0.015	2.24 (2.17, 2.31)	2.29 (2.21, 2.37)	0.001
CA153 (U/mL)	13.49 (9.25, 20.14)	10.4 (8.2, 13.4)	< 0.001	15.72 (10.35, 30.1)	10.14 (8.02, 13.4)	< 0.001
CEA (ng/mL)	4.42 (2.56, 9.99)	2.8 (1.9, 4.1)	< 0.001	8.52 (3.44, 55.06)	2.92 (2.04, 4.6)	< 0.001
CYFRA211 (ng/mL)	5.1 (2.88, 11.03)	2.5 (2, 3.2)	< 0.001	5.67 (2.97, 9.46)	2.53 (1.96, 3.31)	< 0.001
DBIL (umol/L)	4.02 (3.23, 5.26)	4.5 (3.6, 5.7)	< 0.001	4.44 (3.43, 5.93)	4.57 (3.64, 5.69)	0.864
EO# (10^9^/L)	0.1 (0.05, 0.19)	0.1 (0.1, 0.2)	0.037	0.1 (0.05, 0.17)	0.09 (0.05, 0.15)	0.444
Fib (g/L)	3.78 (2.8, 5.26)	2.9 (2.5, 3.4)	< 0.001	3.56 (2.76, 4.73)	2.87 (2.5, 3.48)	< 0.001
GAMMA (%)	17.15 (15.1, 19.6)	16.7 (14.8, 18.7)	0.032	16.8 (14.4, 19.3)	16.5 (14.6, 18.4)	0.577
GLB (g/L)	29.6 (27.2, 33)	28.8 (26.4, 31.9)	0.004	28.7 (26.2, 32.3)	28.5 (26.1, 31.6)	0.702
GLU (mmol/L)	5.37 (4.82, 6.11)	5.3 (4.9, 6)	0.822	5.57 (5, 6.28)	5.45 (4.96, 6.23)	0.455
HCT (%)	40.75 (37.23, 44)	41.2 (38.5, 44.3)	0.033	40.1 (36.5, 42.6)	40.9 (38.4, 44.2)	0.032
HGB (g/L)	132 (120143.75)	135 (126146)	0.01	132 (119140)	134 (125145)	0.008
LYMPH# (10^9^/L)	1.39 (1.08, 1.7)	1.5 (1.2, 1.9)	0.001	1.34 (0.95, 1.73)	1.47 (1.12, 1.93)	0.04
MCV (fl)	91.8 (88.33, 94.8)	92.3 (89.8, 95.1)	0.03	92.1 (89.1, 95)	92.5 (89.9, 95.4)	0.267
MONO# (10^9^/L)	0.52 (0.4, 0.66)	0.4 (0.3, 0.5)	< 0.001	0.53 (0.38, 0.69)	0.4 (0.31, 0.53)	< 0.001
MPV (fl)	10.6 (10, 11.5)	11.2 (10.5, 11.9)	< 0.001	11.1 (10.4, 11.6)	11.2 (10.5, 11.9)	0.347
NEUT# (10^9^/L)	4.45 (3.27, 6.01)	3.6 (2.8, 4.7)	< 0.001	4.45 (3.2, 5.88)	3.7 (2.9, 4.73)	< 0.001
NSE (ng/mL)	19.46 (14.98, 28.05)	16 (13.4, 18.8)	< 0.001	19.79 (14.93, 28.2)	15.87 (13.12, 18.95)	< 0.001
PCT^a^	0.26 (0.22, 0.31)	0.2 (0.2, 0.3)	< 0.001	0.25 (0.2, 0.3)	0.23 (0.2, 0.28)	0.102
PCT^b^ (ng/mL)	2.27 (1.02, 4.35)	1.5 (0.7, 3.2)	< 0.001	4.52 (1.02, 10.48)	1.62 (0.66, 3.88)	< 0.001
PLT (10^9^/L)	245.5 (199302)	213 (172256)	< 0.001	222 (183276)	211 (171255)	0.112
ProGRP (pg/ml)	58.39 (44.09, 82.7)	55 (47, 67.1)	0.089	67.55 (47.9, 89.7)	54.77 (48.5, 65.88)	0.001
PT (s)	10.8 (10.4, 11.49)	10.6 (10.1, 11)	< 0.001	10.9 (10.4, 11.5)	10.5 (10.1, 11)	< 0.001
RBC (10^12^/L)	4.41 (4.08, 4.87)	4.5 (4.1, 4.8)	0.185	4.35 (4.02, 4.69)	4.45 (4.12, 4.81)	0.099
RDW	12.9 (12.3, 13.7)	12.6 (12.2, 13.2)	< 0.001	13 (12.6, 13.6)	12.7 (12.2, 13.2)	< 0.001
SCCA (ng/mL)	1.42 (0.89, 3.06)	1.1 (0.8, 1.4)	< 0.001	1.29 (0.76, 2.53)	1.06 (0.76, 1.35)	0.005
TBIL (umol/L)	14.23 (11.02, 18.12)	16.2 (13.2, 20.8)	< 0.001	15.2 (10.87, 20.65)	16.2 (13.03, 20.62)	0.119
TP (g/L)	68.75 (63.85, 73.6)	71.6 (66.5, 75.4)	< 0.001	66.4 (60.7, 71.5)	70.9 (65.4, 75.1)	< 0.001
TT (s)	16.9 (16.1, 17.68)	17.4 (16.8, 18.1)	< 0.001	16.9 (16.3, 18)	17.4 (16.8, 18.1)	0.01
WBC (10^9^/L)	6.5 (5.34, 8.24)	5.8 (4.9, 7.1)	< 0.001	6.74 (5.41, 8.24)	5.88 (4.87, 7.14)	0.002

Abbreviations: Group M, the skip regional lymph node distant metastasis prediction group; Group N, the regional lymph node involvement prediction group; M−, the non‐metastasis group; M+, the metastasis group; N−, non‐invasive group; N+, invasive group.

### 3.4 Identification of Predictive Model Features

**FIGURE 2:**
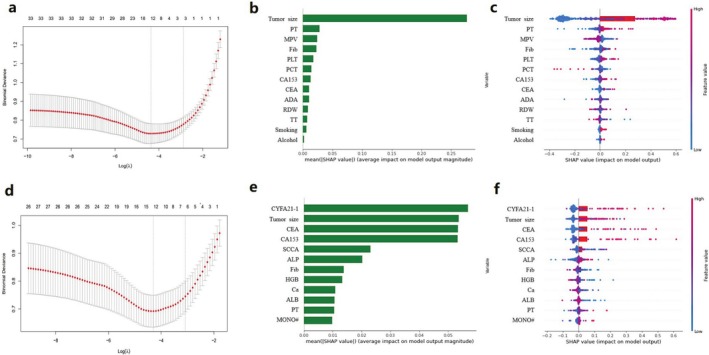
Identification of predictive model features. Identification of predictive model features using LASSO regression. (a, d) Cross‐validation curves for LASSO regression‐based feature selection. (b, e) Bar plots depicting individual feature SHAP values. (c, f) Bee swarm plots illustrating feature values and their influence on model predictions.

Univariate analysis identified significant differences in 40 parameters in Group N and 27 parameters in Group M. The significant parameters were further selected using lasso regression, yielding optimal lambda values with logarithms of 13 and 12 for Groups N and M, respectively. Consequently, 13 and 12 variables were selected as predictive indicators for further analysis. Specifically, the selected variables for Group N included tumor size, PT, MPV, Fib, PLT, PCT^b^, CA153, CEA, ADA, RDW, TT, smoking history, and alcohol consumption history. The selected variables for Group M included cyfra211, tumor size, CEA, CA153, SCCA, ALP, Fib, HGB, Ca, ALB, PT, and MONO#. The construction of these predictive models is visually illustrated in Figure 2.

### 3.5 Model Construction and Validation

The selected variables were used to build the ML models, and the test cohort was used to evaluate the performance of the established prediction models. In the ML algorithms, regional lymph node invasion and distant metastasis were considered as the positive category, while the absence of metastasis was considered as the negative category. Logistic regression, random forest (RF), XGBoost, and AdaBoost were used to construct models in both groups.

**FIGURE 3:**
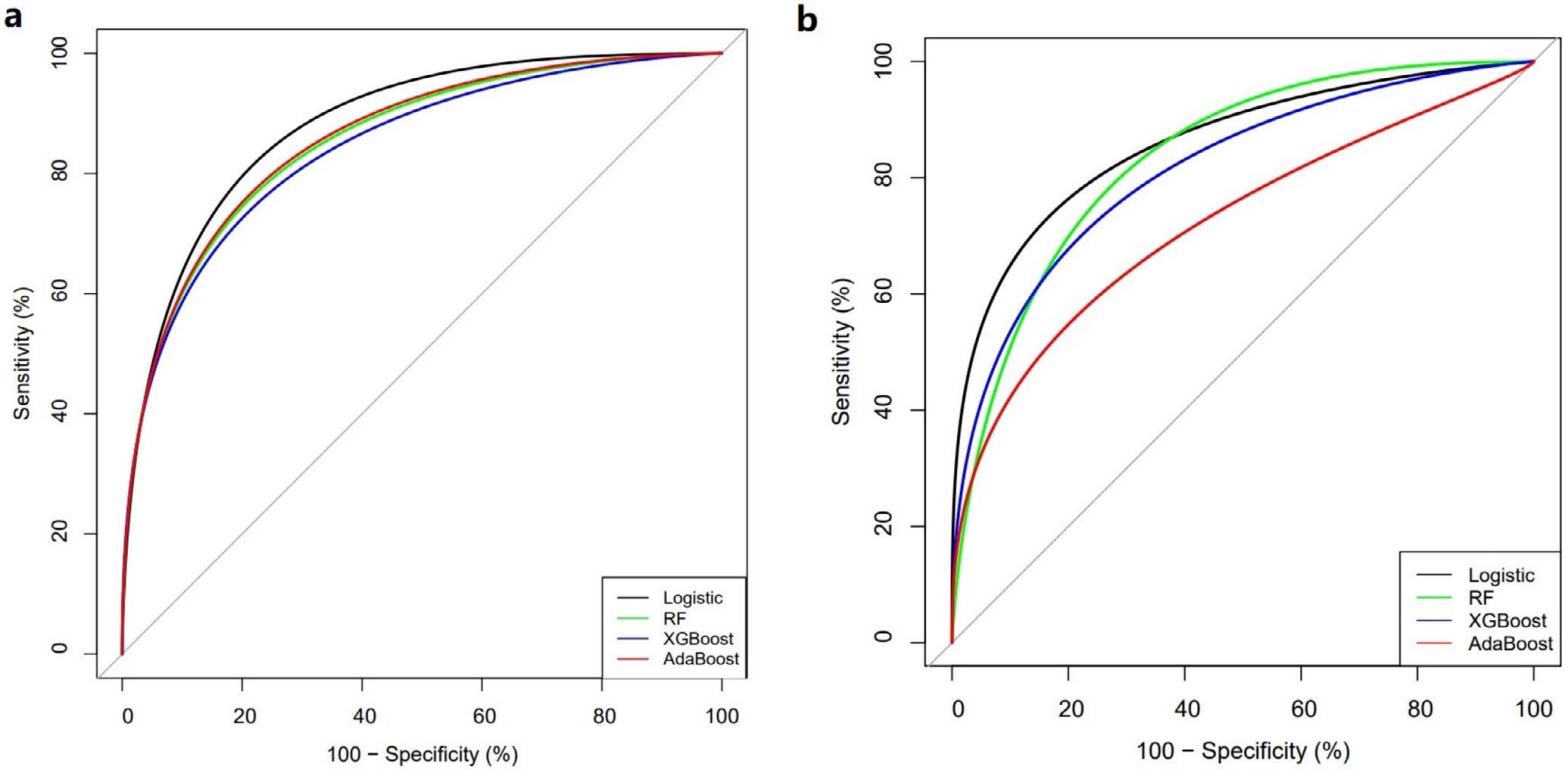
Receiver operating characteristic (ROC) curve analysis for the test cohort of the machine learning algorithm prediction model. Assessment of Model Discrimination via ROC AUC for Various Machine Learning Algorithms. (a) For N group, the AUC results of the test cohort ROC curves for the four ML algorithms were 0.888, 0.854, 0.846, and 0.858, respectively; (b) For M group, the AUC results of the test cohort curves for the four ML algorithms were 0.875, 0.825, 0.806, and 0.725, respectively.

In Group N, the sensitivity of the prediction models in the test cohort was 0.859, 0.803, 0.718, and 0.718 for logistic regression, RF, XGBoost, and AdaBoost, respectively. The specificity was 0.809, 0.771, 0.878, and 0.894, respectively. The discriminant ability of the prediction models was evaluated using the ROC curve, with AUC values of 0.888, 0.854, 0.846, and 0.858 in the test cohort, respectively (Table 5 and Figure 3).

**TABLE 5:** Evaluation of machine learning models on the test cohort.

Model type	Group N				Group M			
	Logistic	RF	XGBoost	AdaBoost	Logistic	RF	XGBoost	AdaBoost
AUC	0.888	0.854	0.846	0.858	0.875	0.825	0.806	0.725
AUC.low	0.844	0.802	0.786	0.807	0.784	0.71	0.678	0.564
AUC.up	0.932	0.907	0.906	0.909	0.966	0.941	0.933	0.885
ACC	0.822	0.78	0.834	0.846	0.878	0.763	0.878	0.801
SEN	0.859	0.803	0.718	0.718	0.75	0.812	0.625	0.625
SPE	0.809	0.771	0.878	0.894	0.893	0.757	0.907	0.821

Abbreviations: ACC, accuracy; AdaBoost, adaptive boosting; AUC, area under the curve; Group M, the skip regional lymph node distant metastasis prediction group; Group N, the regional lymph node involvement prediction group; Logistic, logistic regression; RF, random forest; SEN, sensitivity; SPE, specificity; XGBoost, extreme gradient boosting.

In Group M, the sensitivity of the prediction models in the test cohort was 0.75, 0.812, 0.625, and 0.625 for logistic regression, RF, XGBoost, and AdaBoost, respectively. The specificity was 0.893, 0.757, 0.907, and 0.821, respectively. The discriminant ability of the prediction models was evaluated using the ROC curve, with AUC values of 0.875, 0.825, 0.806, and 0.725 in the test cohort, respectively (Table 5 and Figure 3).

Additionally, we calculated the SHapley Additive exPlanations (SHAP) values for each predictive variable (Figure 2). The higher the SHAP value of a predictor, the greater its contribution to the model. Consequently, the importance of predictors to the model is ranked as follows: for Group N, tumor size, PT, MPV, Fib, PLT, PCT^b^, CA153, CEA, ADA, RDW, TT, smoking history, and alcohol consumption history. For Group M, the predictors are ranked as follows: CYFRA211, tumor size, CEA, CA153, SCCA, ALP, Fib, HGB, Ca, ALB, PT, and MONO#.

### 3.6 Comparison of Previous Relevant Studies

In recent years, studies predicting lymph node metastasis have revealed that the utilized data types include radiomics data, clinical data, and clinical laboratory data, among others. Notably, there is a predominance of single‐center studies in this domain. For the extraction of imaging features, professional personnel are required to assist in the process, and the resulting parameter set is extensive. The number of parameters incorporated in these models varies from 3 to 24, and their prediction efficiencies range from 0.7 to 0.94. In contrast, the data types of the parameters included in this study are simpler and more readily obtainable, yet the prediction efficiency of this model is superior to or comparable with that of most existing studies (Table 6).

**TABLE 6:** Research on lymph node metastasis of lung cancer in recent years.

Year	Publication	Model prediction	Main feature types	Sample size	Total number of extracted imaging features	Feature selection	Parameters	Center category	Modeling method	Performance (AUC)
2020	Mengdi Cong et al. [24]	Lymph node metastasis in NSCLC	CT	411	1229	LASSO	10	Single‐center	LR	0.73
2021	Yunming Xie et al. [25]	Lymph node staging in NSCLC	[^18^F]FDG PET/CT	124	1472	mRMR, LASSO	14	Single‐center	Nomogram	0.872
2022	Xingxing Zheng et al. [20]	Lymph node metastases in NSCLC	CT	217	197	mRMR, LASSO	5	Single‐center	Nomogram	0.7
2022	Boris Gorodetski et al. [26]	Lymph node metastases in lung cancer	CT	381	/	FSM, Wilcoxon, AUC, MI, MRMI	24	Single‐center	LASSO, PLS	0.87
2022	Jieqin Lv et al. [27]	Lymph node metastasis in clinical stage T1 lung adenocarcinoma	PET/CT	183	974	LASSO	12	Single‐center	LR	0.75
2023	Julian M. M. Rogasch et al. [28]	Lymph node metastases in NSCLC	[^18^F]FDG PET/CT	491	/	GBM	10	Single‐center	RF, SVC, GBM, XGB, MLP	0.94
2023	Dai Meng et al. [29]	Mediastinal lymph node metastasis in lung adenocarcinoma	[^18^F]FDG PET/CT	288	/	LR	3	Single‐center	LR	0.849
2024	Nan Meng et al. [30]	Lymph node status in NSCLC	[^18^F]FDG PET/CT	145	2349	Mann–Whitney U test, LASSO, SelectKBest	16	Single‐center	RF, GP, PLS‐DA, QDA, SVM	0.791
2024	HaoJi Yan et al. [31]	Subcarinal lymph node metastasis in NSCLC	CT	202	1427	Boruta, IG, Relief, LASSO, RFE, and RF	3 ~ 14	Single‐center	SVM, KNN, NB, RF, ANN	0.88
2024	Xu Jiang et al. [32]	Occult lymph node metastasis in SCLC	CT	242	1595	LASSO, LR	5	Multicenter	Nomogram	0.772

Abbreviations: [^18^F]FDG PET/CT, ^18^F‐Fluorodeoxyglucose positron emission tomography/CT; 3D‐UTE, three‐dimensional ultrashort echo time; ANN, artificial neu ral network; AUC, Area under the curve; CT, computed tomography; FSM, Feature selection method; GBM, gradient boosting classifier; GP, Gaussian process; IG, information gain; KNN, k‐nearest neighbors; LASSO, least absolute shrink age and selection operator; LR, logistic regression; MI, mutual information; MLP, multi‐layer perceptron classifier; MRMI, maximum relevance minimum redundancy; mRMR, minimum redundancy maximum relevance; NB, naive bayes; NSCLC, nonsmall cell lung cancer; PET, positron emission tomography; PLS, partial least squares; PLS‐DA, partial least squares discriminant analysis; QDA, quad ratic discriminant analysis; RF, random forest; RFE, recursive feature elimination; SCLC, small cell lung cancer; SVC, support vector classifier; SVM, support vector machine; XGB, XGBoost classifier.

## 4 Discussion

Clinical laboratory data provide valuable insights into the growth and metabolic activities of cancer cells, with changes in these markers often indicating the spread of cancer cells to nearby and distant tissues. Univariate analysis identified 40 parameters in the N group and 27 parameters in the M group that demonstrated statistically significant differences (*p* < 0.05) in predicting regional lymph node involvement and skip regional lymph node distant metastasis, respectively. However, the large number of parameters complicates clinical application, highlighting the need for parameter optimization. Using LASSO regression, the study distilled these parameters to 13 factors for the N group and 12 factors for the M group. The patients' clinical characteristics demonstrate that tumor size plays a crucial role, suggesting that larger tumors are more prone to regional lymph node invasion, potentially leading to distant metastasis [37]. Additionally, although a history of smoking and alcohol consumption is associated with regional lymph node involvement, no clear association with distant metastasis was observed.

There is a significant correlation between coagulation‐related parameters and tumor invasiveness in the N group. Previous research has identified prothrombin time (PT) [19], mean platelet volume (MPV) [21], platelet count (PLT) [21], fibrinogen (Fib) [18], and thrombin time (TT) [38] as predictive indicators of lung cancer progression. Additionally, biomarkers such as procalcitonin (PCT) [17], carbohydrate antigen 15–3 (CA153) [39, 40], carcinoembryonic antigen (CEA) [39, 40], adenosine deaminase (ADA) [23], and red blood cell distribution width (RDW) [20] have shown clinical relevance in lung cancer metastasis. This study further highlights significant differences in these laboratory parameters between groups with and without regional lymph node invasion in lung cancer (*p* < 0.05), indicating their potential as powerful predictors of lung cancer invasiveness.

Lung cancer can demonstrate skip regional lymph node metastasis to distant lymph nodes during its invasive progression [6, 12, 13]. It is a critical event that categorizes lung cancer as stage IV, significantly influencing patient survival rates and necessitating a shift from curative to palliative treatment strategies [6, 41]. The study found that CEA, CA 15–3, Fib, and PT are not only predictors for regional lymph node involvement but also have significant predictive value for skip regional lymph node metastasis in lung cancer. The study included key parameters such as Cytokeratin 19 Fragment (CYFRA 21‐1) [40], squamous cell carcinoma antigen (SCCA) [42, 43, 44], Hemoglobin(HGB) [45], Calcium(Ca) [46], Albumin(ALB) [47, 48], and absolute monocyte count (MONO#) [15, 16], all known to be closely related to lung cancer prognosis. The research findings validated the connection of these indicators to distant metastasis in lung cancer, providing a solid basis for assessing prognostic biomarkers.

Radiology plays a crucial role in assessing lung cancer metastasis, with each imaging modality offering unique advantages and limitations [7]. The rapid progress of machine learning (ML) has enabled the integration of diverse clinical data, offering a new perspective on lung cancer diagnosis and management [49, 50, 51]. This study used LASSO regression to identify the optimal feature subset and developed a predictive model using four ML algorithms. Our results indicate that in groups N and M, logistic regression outperformed other algorithms, achieving AUC values of 0.888 and 0.875, respectively. The clinical efficacy of this model was superior to or comparable with that of most studies (see Table 6 for details). When the complexity of the model parameters is significantly lower than that of the imaging parameters, this study achieves a more straightforward and accurate research objective. This difference may be due to the limited sensitivity of imaging techniques in detecting smaller metastatic lesions [52], while blood‐based biomarkers may better reflect lung cancer progression [53]. However, specific models, such as the one by Jieqin Lv et al. [27] on PET/CT characteristic parameters, demonstrated higher diagnostic efficiency with an AUC of 0.94. Further exploration into subtypes and other aspects is recommended in future studies.

This study has several limitations. First, this study is a single‐center retrospective study. Test results from different laboratories may vary due to differences in detection systems, and results may differ when applied in other centers. Second, the grouped data in this study do not match on a 1:1 basis, and the sample size is limited, which may introduce bias in the analysis results. Future research should consider multicenter studies and appropriate sample size balancing to address these limitations. Additionally, regional lymph node invasion and distant metastasis were primarily identified through various imaging examinations, and some small invasions may not be detected, which could affect the study results. The subsequent phase of the research will focus on further refining the model and conducting external validation across multiple centers to enhance its clinical applicability and generalizability. Simultaneously, it is crucial to initiate long‐term follow‐up studies to assess its efficacy over an extended duration.

## 5 Conclusions

This study demonstrates the potential of using ML algorithms alongside clinical characteristics and laboratory data to predict regional lymph node involvement and skip regional lymph node metastasis in lung cancer. The research findings reveal a new set of biomarkers that may be valuable for clinical applications. The Logistic regression model stands out for its strong diagnostic abilities and warrants more research and real‐world application in clinical settings. Therefore, it is crucial for clinicians to emphasize the analytical value of laboratory data in developing personalized treatment plans for patients.

## Author Contributions

**Chao Du** and **Qi Liu:** conceptualization, writing – original draft. **Chao Du** and **Jun Gong:** methodology, formal analysis, visualization. **Chao Du** and **Changchun Niu:** resources. **Chao Du, Qi Liu, Yuanyuan Guo, Jun Gong, Ling Yan** and **Zhijie Li:** data curation. **Chao Du, Qi Liu** and **Changchun Niu:** writing – review and editing. **Changchun Niu:** supervision, funding acquisition. All authors had full access to the data in the study and take responsibility for the integrity of the data and the accuracy of the data analysis.

## Conflicts of Interest

The authors declare no conflicts of interest.

## Data Availability

Data sharing not applicable to this article as no datasets were generated or analysed during the current study.
